# Mind-wandering dynamics during individual vs. group mindfulness meditation

**DOI:** 10.3389/fpsyg.2026.1912263

**Published:** 2026-08-28

**Authors:** Alessio Matiz, Sara Sorella, Giuseppe Pagnoni, Franco Fabbro, Cristiano Crescentini

**Affiliations:** 1 Department of Human Sciences, Link Campus University, Rome, Italy; 2 Department of Languages and Literatures, Communication, Education and Society, University of Udine, Udine, Italy; 3 Department of Biomedical, Metabolic and Neural Sciences, Section of Physiology and Neuroscience, University of Modena and Reggio Emilia, Modena, Italy; 4 Institute of Mechanical Intelligence, Sant'Anna School of Advanced Studies, Pisa, Italy

**Keywords:** meditation setting, mindfulness meditation, mind-wandering, mood, social effects

## Abstract

**Introduction:**

Although meditation practice encompasses both intrapersonal and interpersonal dimensions, the differential effects of individual versus group meditation settings (IS vs. GS) remain largely unexplored. This study aimed to reduce this gap by comparing the effects of a mindfulness meditation session performed in the two settings, focusing in particular on mind-wandering (MW) dynamics.

**Methods:**

Expert meditators (*n* = 30) performed two 30-min breath-focused mindfulness meditation sessions, one in IS and one in GS. During the meditations, participants reported the occurrence of MW via button presses. After each meditation, participants completed questionnaires assessing state mindfulness, mood, and meditation-related experiences. Trait-like questionnaires were also collected.

**Results:**

Across the two meditation settings, the average number of MW self-reports was similar, but their temporal course was significantly different: the rate of MW reports per time remained almost constant over time in IS, whereas it slightly increased in GS. Moreover, lower scores were observed in GS than in IS for the mood states of tension–anxiety and fatigue–inertia.

**Discussion:**

The present study showed that the temporal dynamics of MW during mindfulness meditation differed depending on the meditation setting. The study also showed that, on average, meditating in a group allowed expert mindfulness practitioners to feel less tired/inert and less tense/anxious at the end of the session compared with meditating individually. Although preliminary, these findings hint at why complementing individual mindfulness practice with group sessions may be useful, for example, when practitioners experience difficulties maintaining a regular practice.

## Introduction

Meditation is an umbrella term for a family of contemplative practices that can modulate psychological and physiological processes (Matko and Sedlmeier, 2019). Similar to other human activities, meditation practices encompass both intrapersonal and interpersonal dimensions. On one side, meditation typically requires practitioners to direct their attention inward, that is, to their moment-by-moment subjective experience. On the other side, during the learning phase, novices often practice meditation—typically alongside peers—while receiving instructions from another person. Moreover, after completing a training program, individuals can perform their meditations either in isolation or together with a group of practitioners. Within the Buddhist tradition, wherein mindfulness meditation originates, the role of the community of practitioners in sustaining individual practice is recognized through the so-called “Sangha effect” (Gunarantana, 1992; Nhat Hanh, 1998). Although meditation research has flourished, in particular for mindfulness meditation, and has highlighted its potential to promote mental health (e.g., Sedlmeier et al., 2012; Galante et al., 2023), it has largely overlooked distinguishing the effects obtained during meditations in individual settings (IS) from those experienced during meditations in group settings (GS). A couple of studies have described the differential effects of a 6/8-week mindfulness training delivered in standard formats—with 6/8 weekly group meetings with the instructor—or through only an initial group meeting with the instructor (Mantzios and Giannou, 2014; Matiz et al., 2018). During GS trainings, however, participants are likely to perform more IS meditations than GS meditations, as they are required to perform daily IS sessions at home between the two GS sessions held one week apart. Therefore, this does not allow to fully capture the effects of IS vs GS meditations. Another set of studies reported differences in brain activity measured through EEG during paired vs IS mindfulness meditation (Matiz et al., 2021; Engelbregt et al., 2021), although the group size was limited to just two meditators. The topic of IS vs GS is increasingly relevant today, with many meditation sessions and trainings delivered online through synchronous GS, audio/video recordings, or apps.

To the best of our knowledge, no study has yet compared the effects of a single mindfulness session performed in IS vs GS. Beyond the differences observed in GS/IS mindfulness trainings and paired/IS mindfulness practices, we hypothesize that differences between GS and IS mindfulness practices may emerge for the following reasons: there are general facilitation or inhibition effects of social contexts on task performance (Aiello and Douthitt, 2001); individual preferences were observed for internet, IS, or GS of mindfulness meditation interventions (Wahbeh et al., 2014); higher frequency of mystical/transcendent and extraordinary experiences during IS versus GS were reported by a large group of meditators (Vieten et al., 2018); differences were observed in state mindfulness and social connection during individual mindfulness practices performed in solitary/group/nature video-simulated meditation practice environments (Hanley et al., 2022).

A study on IS and GS meditations should primarily assess state mindfulness, as it is the primary outcome of a mindfulness practice (Lau et al., 2006). Moreover, positive and negative meditation experiences should also be assessed, for example, through the recently developed Inventory of Meditation Experiences (Van Dam et al., 2024). Another assessment should involve post-meditation mood, given the recognized effects of mindfulness practices on emotion-related outcomes (Hoge et al., 2019). Finally, the assessment of mind-wandering (MW) could be included, since the task of mindfulness meditation is to focus mindfully on the meditation object, recognize MW whenever it arises, and return to the meditation object with reduced reactivity (Feruglio et al., 2021). Moreover, it is known that the being with others can increase arousal (Zajonc, 1965) and this may influence attentiveness and MW in GS. Since MW occurs intermittently during meditation, analyzing its occurrence over time can provide valuable information about the temporal dynamics of a meditation practice. Similar to the single-subject analysis of fMRI recordings during mindfulness meditation (Hasenkamp et al., 2012), MW can be self-reported by participants through a button press whenever they realize their mind has wandered, in the so-called self-caught MW detection paradigm.

The present study investigated the differences between mindfulness meditation practiced by the same participants once in GS and once in IS. Participants included 30 expert meditators, all trained in the same mindfulness program. The meditation task was a 30-min breath-focused mindfulness practice during which participants reported their MW through a button press. After each meditation, participants completed questionnaires on state mindfulness, state mood, and meditation-related experiences that occurred during or immediately after the task. Questionnaires on trait MW were also collected, along with other self-reports on mindfulness-related and general psychological constructs.

## Methods

### Participants

Thirty participants (20 female, age 27–72 years, *M* = 53.57 ± 11.52) took part in the study. They were recruited from individuals who had completed the Mindfulness-Oriented Meditation (MOM) program (Fabbro and Crescentini, 2017; Matiz et al., 2018, 2025) at least 2 years prior to study enrollment. They reported 6.03±3.69 years of mindfulness meditation practice on average. Regarding education level, 5 participants were undergraduates, 20 graduates, and 5 post-graduates. Regarding within-group familiarity, participants, on average, knew by name 4.53 ± 3.21 other group members, and had visited the home of 0.40 ± 0.62 others.

The study was performed in accordance with the 1964 Declaration of Helsinki and approved by the Institutional Review Board of the Department of Languages and Literatures, Communication, Education and Society of the University of Udine [CGPER-2022-12-22-02]. All participants provided informed consent.

### Procedure

The main part of the study took place on a single day (Saturday, May 3, 2025) in a building at the University of Udine in Udine, Italy.

All participants performed two meditation sessions in a within-subject crossover design: one in GS and one in IS. Both sessions consisted of a 30-min breath-focused mindfulness meditation (*anapanasati*). The group meditation for all 30 participants was held at 12:30 p.m. Individual meditations were scheduled before (8:30 a.m.−12:00 p.m.) or after (2:00–4:30 p.m.) the group session, 15 participants per slot, across five separate rooms, each led by an experimenter. Participants practiced silent meditation with eyes closed and no audio/video instructions during the task. All meditations took place in quiet rooms. However, white noise was played during both IS and GS (from the PC and room speakers, respectively) to minimize the influence of potential environmental sounds. Sociodemographic and meditation-related information was collected before the IS meditation.

During the meditation, participants reported their MW by pressing a button on a device they held in one hand. They pressed it whenever they noticed their attention had drifted away from breath sensations. The button device was wired to an *ad-hoc* circuit board connected to a PC, where the times of the button presses were recorded with millisecond precision from 30 button devices in GS and one in IS.

After the meditation task, participants completed the following questionnaires assessing their post-meditation state. The Toronto Mindfulness Scale (TMS; Lau et al., 2006) is a 13-item measure of state mindfulness related to the experience during the meditation, with two subscales, Curiosity (an attitude of wanting to learn more about one's experiences; ω = 0.858) and Decentering (the ability to observe thoughts and feelings in a wider field of awareness without identifying with them; ω = 0.625). The Inventory of Meditation Experiences (IME; Van Dam et al., 2024) is a 30-item questionnaire assessing a broad range of meditation-related experiences occurring during or immediately after the meditation, including unusual, beneficial and potentially adverse experiences. The IME aims to capture both the intensity of each experience and its subjective valence and comprises three subscales: self- and reality-related Distortions (alterations in the sense of self, body, space and time, as well as cognitive and perceptual shifts; ω = 0.874), Enabling Experiences (experiences typically appraised as beneficial or growth-promoting, including feelings of love, connection, insight, emotional balance and mystical or anomalous experiences; ω = 0.873) and Disabling Experiences (experiences that may interfere with psychological, somatic or social functioning, such as anxiety, agitation, distractibility, disturbing memories, reduced motivation or difficulties in relating to others; ω = 0.639). The Profile of Mood States (POMS; McNair et al., 1971; Italian version: Farnè et al., 1991) is a 58-item measure of negative and positive mood states experienced after the meditation, including Tension-Anxiety (ω = 0.688), Anger-Hostility (ω = 0.690), Vigor-Activity (ω = 0.930), Fatigue-Inertia (ω = 0.690), Depression-Dejection (ω = 0.783), and Confusion-Bewilderment (ω = 0.824).

Two days later, participants received an online form to be completed at home, including trait questionnaires: the 5-item Mind Wandering Questionnaire (MWQ; Mrazek et al., 2013; ω = 0.876); the 24-item Five Facet Mindfulness Questionnaire - short form (FFMQ-SF; Bohlmeijer et al., 2011; Italian version: Iani et al., 2020), measuring observe (ω = 0.565), describe (ω = 0.711), act with awareness (ω = 0.856), non-judge (ω = 0.841), and non-react (ω = 0.761); the 16-item Equanimity Scale (ES-16; Rogers et al., 2021), measuring experiential acceptance (ω = 0.856) and non-reactivity (ω = 0.782); the 20-item Social Connectedness Scale—Revised (SCS-R; Lee et al., 2001; Italian version: Capanna et al., 2013; ω = 0.921), measuring the subjective sense of interpersonal closeness and connectedness; the 14-item Mental Health Continuum-Short Form (MHC-SF; Keyes, 2009; Italian version: Petrillo et al., 2015), measuring psychological (ω = 0.852), emotional (ω = 0.825), and social wellbeing (ω = 0.871). Other trait questionnaires were collected in the context of a larger project on the same topic. Questionnaire scores (Table 1) were descriptively compared with validation samples (Italian versions where available), indicating that the sample is consistent with standard populations, with mean scores ranging within ± 1 SD from the validation means.

**Table 1:** Descriptives of questionnaires of trait characteristics.

Measure	Mean and SD of validation sample	Mean	SD	Min	Max
SCS-R	91; 13.83	88.267	12.969	63	115
MHC-SF.EWB	3.97; 1.12	3.733	0.666	2.0	5.0
MHC-SF.SWB	2.73; 1.01	2.553	0.882	1.2	4.2
MHC-SF.PWB	4.45; 1.02	3.647	0.745	1.5	5.0
ES-16.ACC^1^	29.59; 5.71	29.707	4.320	19.2	36.8
ES-16.NR^1^	29.17; 5.99	30.933	4.657	22.4	39.2
MWQ^1^	3.68; 1.01	2.933	0.663	1.6	4.2
FFMQ-SF.OB	15.74; 2.96	15.933	2.053	11.2	20
FFMQ-SF.DE	17.49; 3.47	18.300	2.879	12	23
FFMQ-SF.AA	19.04; 3.66	17.333	2.928	11	23
FFMQ-SF.NR	14.54; 3.75	16.200	2.413	11	21
FFMQ-SF.NJ	14.95; 3.89	17.767	3.380	10	24

SCS-R, social connectedness scale—revised; MHC-SF, mental health continuum—short form; EWB, emotional wellbeing; SWB, social wellbeing; PWB, psychological wellbeing; ES-16, equanimity scale; ACC, experiential acceptance; NR, non-reactivity; MWQ, mind wandering questionnaire; FFMQ-SF, five facet mindfulness questionnaire—short form; OB, observe; DE, describe; AA, act with awareness; NR, non-react; NJ, non-judge. ^1^No Italian validation available.

### Statistical analyses

GS-IS differences in button presses (MW reports) and post-meditation scores (TMS, IME, and POMS) were tested using paired Fisher permutation tests using 1,00,000 random sign-flipping permutations. This was chosen because most paired-difference distributions were non-normal.

To analyze the MW time course, button presses were first examined for erroneous double presses corresponding to single MW occurrences. Consecutive presses were retained only when separated by at least 4s. We empirically considered 2s to be the typical duration of a “long” button press and 4s the maximum duration of two consecutive long button presses made erroneously in response to a single MW event. 4 s were also considered as the interval between pressing a button that signals awareness of MW and the conclusion of the transition to returning to concentration on the breath, in line with previous studies (Hasenkamp et al., 2012). Button presses were then aggregated into consecutive 15-s interval bins across the 30-min sessions, for a total of 120 bins. The outcome variable was the number of button presses per bin. To test whether the temporal evolution of MW differed between IS and GS meditation, the number of presses per bin was analyzed using a linear mixed-effects model. Fixed effects were time (in minutes, midpoints of each 15-s bin), setting (individual vs. group; IS vs. GS), and their interaction. Random effects included random intercepts and random slopes for time by participant.

The time × setting interaction was assessed by comparing the full model including the interaction term to the reduced model without the interaction term, using a parametric bootstrap likelihood-ratio test with 1,000 simulations (PBmodcomp function, pbkrtest package).

Setting-specific slopes and their difference were estimated from the fitted model, with 95% CIs. The analysis included 7,080 bin-level observations from the 30 participants. Simulation-based power for the time × setting interaction was estimated using the fitted mixed-effects model (simr package).

Exploratory correlations were computed between questionnaire scores assessing post-meditation state and button presses in IS and GS. The analyses were performed using the non-parametric Kendall's tau coefficients, given the non-normal distribution of different variables (including number of presses in IS and GS meditations). Kendall's tau-b, which accounts for tied ranks, was preferred to Spearman's rho because it has been shown to provide adequate control of Type I error, minimal bias, tighter confidence intervals, and clearer interpretation in non-normally distributed rating-scale data (Arndt et al., 1999).

Data analyses were conducted in R (version 4.5.0; https://www.R-project.org/) and RStudio (version 2024.12.1 + 563; https://www.rstudio.com/), while questionnaire analyses were performed in JASP (version 0.18; https://jasp-stats.org).

## Results

A paired Fisher permutation test revealed no significant differences between button presses in GS (*M* = 25.37 ± 19.49) and IS meditations (*M* = 24.17 ± 18.94), mean difference (GS—IS) = 1.20, *p* = 0.564.

**Figure 1:**
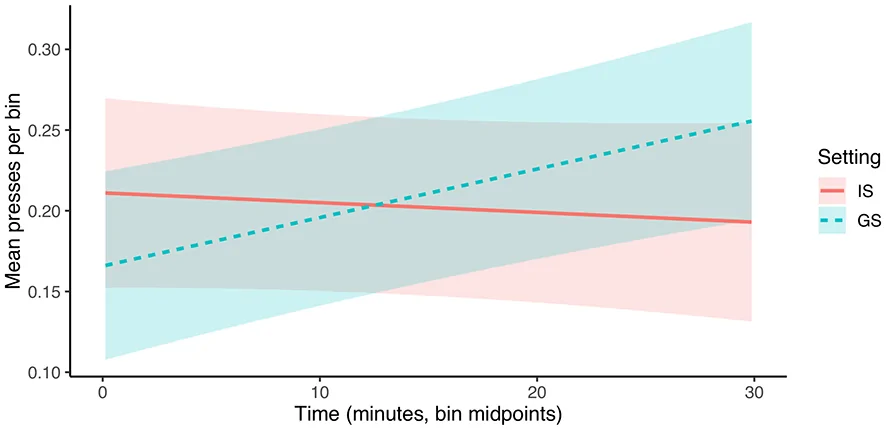
Model-based time course of button presses (i.e., MW reports) during meditation in the IS and GS settings. Lines represent estimated marginal means from the linear mixed-effects model; shaded areas indicate 95% confidence intervals. The GS shows an increasing trend over time, whereas the IS remains stable.

Time course analysis (Figure 1) showed a significant time × setting interaction, assessed by comparing a model including the interaction term to a reduced model without the interaction using a parametric bootstrap likelihood-ratio test (LRT = 10.947, bootstrap *p* = 0.003; 1,000 simulations), indicating different temporal dynamics across settings. The estimated slope difference (GS—IS) was 0.0036 presses per bin per minute (SE = 0.0011, *z* = 3.331, *p* = 0.001), indicating that for each additional minute from the start of meditation, the predicted GS—IS difference increased by 0.0036 presses per bin. Expressed as presses per minute, this corresponds to an increase of approximately 0.014 presses/min for each additional minute, or approximately 0.43 presses/min from the beginning to the end of the 30-min session. The estimated slope in IS was close to zero [*b* = −0.0006, SE = 0.0009, 95% CI (−0.0023, 0.0011)], whereas the slope in GS showed a positive temporal trend [*b* = 0.0030, SE = 0.0009, 95% CI (0.0013, 0.0047)]. Simulation-based power to detect the observed time × setting interaction with the present sample size was 91.9%, 95% CI [90.03%, 93.52%], based on 1,000 simulations. The time × setting interaction remained significant after including condition order (GS-first vs. IS-first) as an additional fixed effect (LRT = 10.935, *p* = 0.001).

Exploratory comparisons between questionnaires after GS and IS meditations revealed a significant difference in the POMS subscales of Tension-Anxiety (mean difference = −0.93, *p* = 0.046, repeated-measures *d* = −0.326) and Fatigue-Inertia (mean difference = −0.83, *p* = 0.032, repeated-measures *d* = −0.278), showing higher IS scores (Table 2). Admittedly, these results would not survive correction for multiple comparisons and should therefore be interpreted as preliminary trends that warrant replication.

**Table 2:** Paired Fisher permutation tests comparing button presses and post-meditation questionnaires between GS and IS meditations.

Group setting (GS)	Individual setting (IS)	Mean difference (GS—IS), 95% CI	*p*	repeated measures Cohen's *d*
Number of presses (25.37; 19.49)	Number of presses (24.17; 18.94)	1.200 [−2.633, 5.067]	0.564	0.061
TMS. Curiosity (13.10; 4.64)	TMS. Curiosity (13.17; 5.90)	−0.067 [−1.532, 1.267]	0.963	−0.012
TMS. Decentering (18.80; 4.01)	TMS. Decentering (18.4; 3.65)	0.400 [−0.767, 1.567]	0.485	0.101
IME. Distortions (13.40; 8.85)	IME. Distortions (12.13; 8.67)	1.267 [−0.100, 2.600]	0.093	0.141
IME. Disabling (6.2; 4.87)	IME. Disabling (7.47; 4.52)	−1.267 [−2.800, 0.133]	0.120	−0.262
IME. Enabling (14.80; 7.44)	IME. Enabling (15.03; 10.24)	−0.233 [−2.733, 1.833]	0.828	−0.023
POMS. Tension-Anxiety(2.47; 2.01)	POMS. Tension-Anxiety (3.4; 3.15)	−0.933 [−2.133, −0.200]	0.046^*^	−0.326
POMS. Depression-Dejection (1.57; 2.33)	POMS. Depression-Dejection (1.4; 2.39)	0.167 [−0.733, 0.767]	0.673	0.069
POMS. Anger-Hostility (0.63; 1.27)	POMS. Anger-Hostility (1.10; 2.55)	−0.467 [−1.800, 0.000]	0.196	−0.197
POMS. Vigor-Activity (17.20; 6.19)	POMS. Vigor-Activity (16.27; 7.09)	0.933 [−0.433, 3.467]	0.350	0.135
POMS. Fatigue-Inertia (1.97; 1.94)	POMS. Fatigue-Inertia (2.80; 3.06)	−0.833 [−1.667, −0.233]	0.032^*^	−0.278
POMS. Confusion-Bewilderment (4.47; 3.30)	POMS. Confusion-Bewilderment (4.70; 4.29)	−0.233 [−1.600, 0.633]	0.677	−0.057

Means and standard deviations for each variable are reported in parentheses (M; SD). Mean differences are computed as GS—IS. *p*-values were obtained from paired Fisher permutation tests using 1,00,000 random sign-flipping permutations. Effect size is repeated-measures Cohen's *d* corrected for small-sample bias. GS, group setting; IME, inventory of meditation experiences; IS, individual setting; POMS, profile of mood states; TMS, toronto mindfulness scale. ^*^ = *p* < 0.05.

Exploratory correlations revealed a negative significant correlation between POMS Vigor-Activity and the number of presses during the IS meditation (tau = −0.295, *p* = 0.026), as well as a positive significant correlation between POMS Anger-Hostility and the number of presses during the GS meditation (tau = 0.316, *p* = 0.033) (Table 3).

**Table 3:** Kendall correlations between questionnaire scores and the number of button presses (i.e., MW reports) during IS and GS meditations.

Scale	Statistics	Number of button presses—IS	Number of button presses—GS
TMS. Curiosity	Kendall's Tau B	−0.252	−0.104
	*p*-value	0.059	0.430
TMS. Decentering	Kendall's Tau B	−0.071	−0.189
	p-value	0.600	0.156
IME. Distortions	Kendall's Tau B	−0.085	−0.012
	*p*-value	0.519	0.929
IME. Disabling	Kendall's Tau B	−0.036	−0.093
	*p*-value	0.787	0.484
IME. Enabling	Kendall's Tau B	−0.240	−0.169
	*p*-value	0.072	0.198
POMS. Tension-Anxiety	Kendall's Tau B	0.190	−0.015
	*p*-value	0.162	0.913
POMS. Depression-Dejection	Kendall's Tau B	0.261	−0.006
	*p*-value	0.075	0.967
POMS. Anger-Hostility	Kendall's Tau B	0.164	0.316^*^
	*p*-value	0.268	0.033
POMS. Vigor-Activity	Kendall's Tau B	−0.295^*^	−0.167
	*p*-value	0.026	0.203
POMS. Fatigue-Inertia	Kendall's Tau B	0.055	0.173
	*p*-value	0.688	0.217
POMS. Confusion-Bewilderment	Kendall's Tau B	0.083	0.058
	*p*-value	0.539	0.665

GS, group setting; IME, inventory of meditation experiences; IS, individual setting; POMS, profile of mood states; TMS, toronto mindfulness scale. ^*^ = *p* < 0.05.

## Discussion

This study aimed to evaluate differences between mindfulness meditation performed in GS and IS. To this end, two types of data were collected: (1) the number and temporal distribution of MW episodes occurring during IS and GS, and (2) self-reports of state mindfulness, meditation-related experiences, and mood states experienced during or immediately after each meditation session, as well as questionnaires assessing trait MW and other trait-like measures.

On average, across the two meditation settings, participants reported MW approximately every 72 s, similarly to the original study by Hasenkamp et al. (2012), in which meditators reported MW every 80 s during a 20-min meditation session performed individually. When comparing MW episodes reported in GS and IS, no difference was observed in their number. This suggests that the meditation setting did not influence average MW reports.

However, a significant difference between the two meditation settings was observed in the temporal course of MW episodes. In IS, the number of MW episodes remained almost constant over time, whereas it slightly increased over time in GS. Thus, during the initial part of GS meditation, the number of self-reported MW episodes was lower than during IS, whereas in the final part it was higher. This result can be interpreted in two different ways, depending on whether the self-report of an MW episode, as measured in the current study, is viewed as an index of increased meta-awareness of one's own MW (perspective 1) or as a sign of reduced attentional engagement (perspective 2).

According to the first perspective, the observed effect of setting could be interpreted as suggesting that, compared with meditating individually, meditating in a group impeded meta-awareness during the initial stages of practice and enhances it during the later stages. According to the second perspective, meditating in a group initially enhanced attentional engagement but later increases distractibility. Although both interpretations are plausible, perspective 1 appears to be supported by two considerations. First, the method used here to detect MW (i.e., self-caught MW) requires a greater degree of participant meta-awareness than other methods, such as probe-caught experience sampling methods, questionnaires, or behavioral tasks (Chu et al., 2023; Nazari et al., 2025). Second, the literature shows that MW generally increases in frequency over time during task performance when assessed with probe-caught experience sampling methods (e.g., Zanesco et al., 2025), suggesting that participants were more aware of this process in the GS. Nonetheless, perspective 2 may be linked with the possibility that environmental distractions arising from the physical presence of a group may have progressively disrupted participants' concentration over the 30-min session, even though the white noise played during the meditations was intended to minimize effect of these distractions.

The data collected through questionnaires after GS and IS can help further explore possible interpretations of the temporal course of MW episodes. No differences between settings were observed in terms of state mindfulness or meditation-related experiences. However, differences were found for the mood scales of tension–anxiety and fatigue–inertia, although these effects would not survive correction for multiple comparisons: immediately after GS, these mood scores were lower than after IS. This result suggests that participants were less tense and fatigued during the final part of GS than during the final part of IS meditation. Since MW is known to increase with tension–anxiety and fatigue (e.g., Randall et al., 2014), this finding argues against the interpretation suggested by perspective 2. Moreover, the lack of difference between GS and IS in the disabling subscale of the meditation-related experiences—which includes distractibility—appears in contrast with the hypothesis that participants could become progressively more distracted by environmental sounds during the GS meditation.

A final result of the current study is that, in the GS, the number of self-reported MW episodes was positively correlated with anger–hostility mood scores, whereas in the IS it was negatively correlated with vigor–activity mood scores. Anger–hostility is a mood state related to social interaction (Weber, 2004) and would therefore be expected to emerge more in GS than in IS. The result concerning vigor–activity appears to indicate that, at least in the IS, the number of MW reports is linked to how vigorous and active participants felt after meditation. This information cannot directly aid in interpreting the different temporal course of MW reports across the two settings, since the rate of MW reports remained almost constant in IS. However, it confirms that MW reports in the current study were somehow related to moods of vigor-activity, or, on the contrary, fatigue-inertia as shown by the result on the post-meditation mood.

Limitations of the present study include the restricted sample size, the exploratory nature of the analyses, the limited number of measures, and the timing of data collection. The number of thirty meditators was determined by technical constraints of the recording apparatus. Future studies should replicate these findings in larger samples, for example by repeating the experiment on many groups of meditators. It seems also worthwhile to extend this experimental protocol to other samples, which differ from the present one in terms, e.g., of meditation expertise or within-group familiarity, or to assess how outcomes can change over time, e.g. from the beginning to the end of a meditation training period. The second limitation is the exploratory nature of the between-setting tests of post-meditation scores, which have not been corrected for multiple comparisons. Regarding the third limitation, future studies could employ different self-report measures and include third-person measures, which are currently being adopted only in dyadic meditation studies (e.g., Matiz et al., 2021). Finally, although condition order (i.e., GS-first vs. IS-first) was counterbalanced, the fixed midday timing of GS could have introduced time-of-day confounds, such as circadian rhythms, attention/vigilance, pre-/post-lunch fatigue or hunger. Future studies should counterbalance both setting and time of day, ideally scheduling GS and IS at equivalent times on separate days.

In conclusion, this first study on individual and group mindfulness meditations suggests that, on average, meditating in a group allowed expert mindfulness practitioners to feel less tired/inert and less tense/anxious at the end of the session, compared with meditating individually. Although preliminary, these findings hint at why complementing individual mindfulness practice with group sessions may be useful. For example, meditating in group could help when practitioners experience difficulties maintaining a regular practice. Nevertheless, the present results obtained on a group of expert meditators should be extended and tested also on larger samples of less expert meditators or novices to meditation. The study also showed that the temporal dynamics of MW during meditation in the GS were different from those in the IS. Although a speculative interpretation of progressively increasing meta-awareness in the group setting was advanced, this point appears to merit further investigation in order to be fully understood and correctly applied.

## Data Availability

The raw data supporting the conclusions of this article will be made available by the authors, without undue reservation.
